# A numerical study of the hemodynamic behavior and gas transport in cardiovascular systems with severe cardiac or cardiopulmonary failure supported by venoarterial extracorporeal membrane oxygenation

**DOI:** 10.3389/fbioe.2023.1177325

**Published:** 2023-05-09

**Authors:** Wenhao Cui, Tianqi Wang, Zhuoming Xu, Jinlong Liu, Sergey Simakov, Fuyou Liang

**Affiliations:** ^1^ Department of Engineering Mechanics, School of Naval Architecture, Ocean and Civil Engineering, Shanghai Jiao Tong University, Shanghai, China; ^2^ School of Mechanical Engineering, University of Shanghai for Science and Technology, Shanghai, China; ^3^ Cardiac Intensive Care Unit, Department of Thoracic and Cardiovascular Surgery, Shanghai Children’s Medical Center, School of Medicine, Shanghai Jiao Tong University, Shanghai, China; ^4^ Institute of Pediatric Translational Medicine, Shanghai Children’s Medical Center, School of Medicine, Shanghai Jiao Tong University, Shanghai, China; ^5^ Department of Computational Physics, Moscow Institute of Physics and Technology, Dolgoprudny, Russia; ^6^ Marchuk Institute of Numerical Mathematics of the Russian Academy of Sciences, Moscow, Russia; ^7^ State Key Laboratory of Ocean Engineering, School of Naval Architecture, Ocean and Civil Engineering, Shanghai Jiao Tong University, Shanghai, China; ^8^ World-Class Research Center “Digital Biodesign and Personalized Healthcare”, Sechenov First Moscow State Medical University, Moscow, Russia

**Keywords:** venoarterial extracorporeal membrane oxygenation, computational model, hemodynamic behavior, gas transport, oxygen saturation, cardiopulmonary failure

## Abstract

Venoarterial extracorporeal membrane oxygenation (VA-ECMO) has been extensively demonstrated as an effective means of bridge-to-destination in the treatment of patients with severe ventricular failure or cardiopulmonary failure. However, appropriate selection of candidates and management of patients during Extracorporeal membrane oxygenation (ECMO) support remain challenging in clinical practice, due partly to insufficient understanding of the complex influences of extracorporeal membrane oxygenation support on the native cardiovascular system. In addition, questions remain as to how central and peripheral venoarterial extracorporeal membrane oxygenation modalities differ with respect to their hemodynamic impact and effectiveness of compensatory oxygen supply to end-organs. In this work, we developed a computational model to quantitatively address the hemodynamic interaction between the extracorporeal membrane oxygenation and cardiovascular systems and associated gas transport. Model-based numerical simulations were performed for cardiovascular systems with severe cardiac or cardiopulmonary failure and supported by central or peripheral venoarterial extracorporeal membrane oxygenation. Obtained results revealed that: 1) central and peripheral venoarterial extracorporeal membrane oxygenation modalities had a comparable capacity for elevating arterial blood pressure and delivering oxygenated blood to important organs/tissues, but induced differential changes of blood flow waveforms in some arteries; 2) increasing the rotation speed of extracorporeal membrane oxygenation pump (*ω*) could effectively improve arterial blood oxygenation, with the efficiency being especially high when *ω* was low and cardiopulmonary failure was severe; 3) blood oxygen indices (i.e., oxygen saturation and partial pressure) monitored at the right radial artery could be taken as surrogates for diagnosing potential hypoxemia in other arteries irrespective of the modality of extracorporeal membrane oxygenation; and 4) Left ventricular (LV) overloading could occur when *ω* was high, but the threshold of *ω* for inducing clinically significant left ventricular overloading depended strongly on the residual cardiac function. In summary, the study demonstrated the differential hemodynamic influences while comparable oxygen delivery performance of the central and peripheral venoarterial extracorporeal membrane oxygenation modalities in the management of patients with severe cardiac or cardiopulmonary failure and elucidated how the status of arterial blood oxygenation and severity of left ventricular overloading change in response to variations in *ω*. These model-based findings may serve as theoretical references for guiding the application of venoarterial extracorporeal membrane oxygenation or interpreting *in vivo* measurements in clinical practice.

## 1 Introduction

Extracorporeal membrane oxygenation (ECMO) is an advanced technique that provides a bridge to decision or ultimate destination in the treatment of patients suffering from severe systemic hypoxemia caused by cardiac failure or pulmonary failure (King et al., 2017; Lorusso et al., 2021). The clinical use of ECMO can be traced back to 1972 (Hill et al., 1972), and the continuous technical advances in cannulation, oxygenator and other components have greatly promoted the applications of ECMO to wide clinical scenarios in the past decades (Napp et al., 2016), including the treatment of patients with severe acute respiratory distress syndrome (ARDS) caused by COVID-19 in recent years (Bartlett et al., 2020; Ramanathan et al., 2020). The configuration of ECMO circuit can be categorized into two major types according to the locations of cannulation for blood drainage and return, namely, veno-venous ECMO (VV-ECMO) and venoarterial ECMO (VA-ECMO). The former is used mainly to support respiratory function in patients with severe ARDS or pneumonia while preserved cardiac function, whereas the latter is frequently applied to patients with cardiopulmonary failure given its ability to provide both circulatory support and extracorporeal blood oxygenation (Napp et al., 2016).

Despite the well-documented lifesaving role of ECMO, the prevention and management of complications (e.g., vascular injury, bleeding, thrombosis, and hypoxia of important organs) associated with ECMO support continue to be a challenging issue in clinical practice (King et al., 2017; Pillai et al., 2018). In addition, the strategies and techniques for monitoring critical physiological indices or hemodynamic variables during ECMO support remain to be improved in order to better cope with patient-specific pathophysiological conditions (Krishnan and Schmidt, 2019). These issues urge the need to thoroughly understand the interaction of ECMO with the native cardiovascular system. In this context, many *in vivo* studies on patients (Bělohlávek et al., 2010; Ius et al., 2015; Kara et al., 2016) or animals (Golej et al., 2002; Solholm et al., 2020) have been carried out. These studies provided important insights for understanding the effects of ECMO from various perspectives spanning from systemic hemodynamics to microcirculation perfusion, nevertheless, most of them were restricted by available techniques for *in vivo* measurements, making the findings less comprehensive or lack quantitative details on some variables of interest (e.g., flow patterns near the cannulation sites, oxygen saturations in different end-organs). Such limitations of *in vivo* studies may be partly overcome by *in vitro* or *ex-vivo* studies, especially those based on mock loop models (Ki et al., 2019; Fu et al., 2021; Caspari et al., 2022; Rozencwajg et al., 2022; Sayed et al., 2022). This sort of studies are however limited by the high cost of fabricating models mimicking the complex vascular structure and difficulties in representing various cardiovascular properties using artificial devices. Relatively, computational modeling methods offer a cheaper while more flexible approach.

Computational models have been widely employed to address issues related to ECMO. Existing models can be grouped into several families according to the range of modeling, i.e., local models dedicated to simulating flow patterns in the vicinity of cannulation (Gu et al., 2016; Stevens et al., 2017; Conrad and Wang, 2020), regional models focused on quantifying ECMO-induced hemodynamic changes in large arteries (Feiger et al., 2021; Seetharaman et al., 2021), and global models applied to investigate the impact of ECMO on systemic hemodynamics (Colasanti et al., 2021; Lazzari et al., 2021). Moreover, there existed some studies concerned with the transport of oxygenated blood in the cardiovascular system (Zanella et al., 2016; Joyce et al., 2018; Stevens et al., 2018; Feiger et al., 2020; Khodaee et al., 2022). These studies provided quantitative evidence for understanding how the oxygenated blood flowing through the ECMO system mixes with the blood in the native cardiovascular system to alter hemodynamic characteristics or improve the status of blood oxygenation in certain arteries or end-organs of concern. Nevertheless, most studies introduced more or less assumptions/simplifications that may compromise the physiological fidelity or clinical implications of numerical results. For instance, the majority of studies, except for a few ones (Zanella et al., 2016; Joyce et al., 2018), neglected gas exchanges in the lung, microcirculation, and membrane oxygenator that are important for representing pulmonary dysfunction, metabolic state, and properties of ECMO. Regional models established with one-dimensional or three-dimensional modeling methods had the advantage of simulating in detail the transport of ECMO-oxygenated blood and its interaction with the residual native blood flow in the arterial system (Stevens et al., 2018; Feiger et al., 2020; Khodaee et al., 2022), but they usually needed the imposition of a blood flow waveform at the aortic root as the inflow boundary conditions, which essentially ignored the role of the dynamic interaction between the residual cardiopulmonary function and ECMO support in determining the characteristics of systemic hemodynamics and gas transport. The deficiency can be addressed by global models, which were generally established with the lumped-parameter modeling method to integrate the heart, complex systemic and pulmonary vasculatures and ECMO into a unique model framework (Zanella et al., 2016; Joyce et al., 2018; Colasanti et al., 2021; Lazzari et al., 2021). However, lumped-parameter models often adopted a simplified compartmental representation of the vascular system, which could reduce the difficulty of parameter assignment and facilitate flexible simulations of various clinical scenarios, but at the expense of reduced level of detail in delineating hemodynamic behavior and oxygen transport in the arterial system.

In the present study, we developed a computational model capable of describing in detail the transport of oxygen and carbon dioxide in the arterial system whilst accounting for the effects of ECMO support in the context of systemic hemodynamic behavior and gas exchange/transport throughout the whole body. To this aim, we adopted a geometrical multi-scale modeling method wherein a one-dimensional (1D) model is built to provide detailed information of blood flow and gas transport in large arteries, which is further coupled to lumped-parameter (0D) models of other cardiovascular portions, lung and ECMO to yield a closed-loop representation of the entire cardiopulmonary-ECMO system. With the model, a series of numerical simulations were carried out to address several issues that are important for guiding the clinical application of ECMO but remain less explored by previous clinical or numerical studies: 1) how the central and peripheral VA-ECMO modalities differ in terms of their influences on blood flow/pressure and oxygen distribution in the arterial tree; 2) whether blood oxygen indices monitored at the radial artery can indicate the status of blood oxygenation in the perfusion arteries of important organs/tissues; and 3) under what conditions the left ventricle is significantly overloaded by ECMO support?

## 2 Materials and methods

**FIGURE 1:**
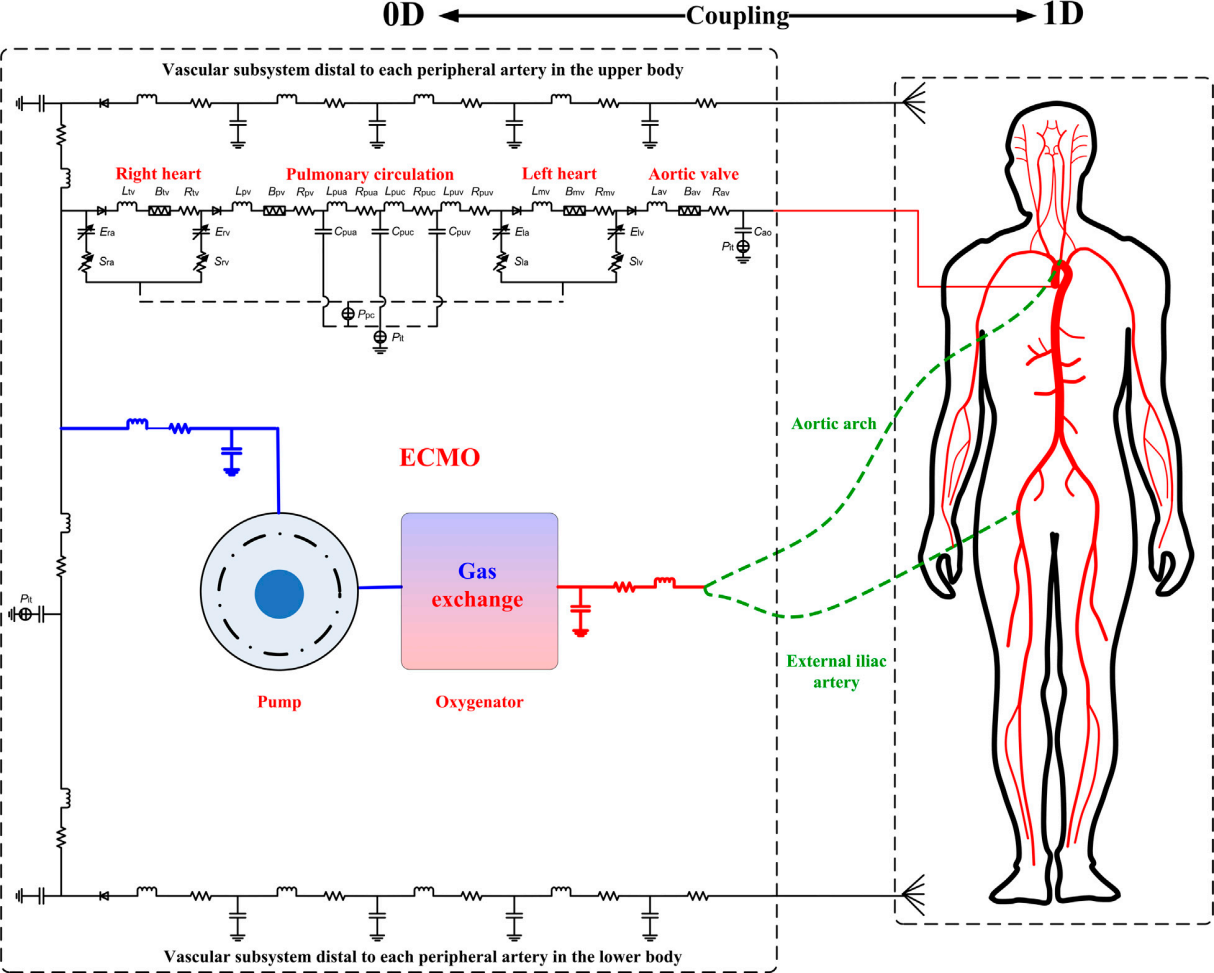
Schematic diagram of the coupled ECMO-cardiovascular model. The cardiovascular model consists of a 1D model of the arterial tree coupled to 0D models of other cardiovascular portions including the heart, systemic vasculatures, and pulmonary circulation. The 0D model of the ECMO system is coupled to the cardiovascular model through connecting the inflow and outflow cannulas to the right atrium and an artery (the aortic arch in the case of central VA-ECMO, whereas the right external iliac artery in the case of peripheral VA-ECMO), respectively. It is noted that the models of gas exchange and transport are not explicitly illustrated, which take hemodynamic variables computed by the hemodynamic model as the inputs to compute gas variables but do not affect blood flow in turn, and hence are solved separately after convergence of hemodynamic simulation (see the text for more detailed explanations).

The computational model consists of two major parts, namely, hemodynamic part and gas exchange and transport part. The hemodynamic part was obtained by incorporating a lumped-parameter model of the ECMO system into a zero-one dimensional (0-1D) multi-scale model of the entire cardiovascular system developed in our previous studies (Liang et al., 2009a; Liang et al., 2009b) (see Figure 1). In the hemodynamic model, large arteries were represented by a distributed 1D model, which was coupled to a lumped-parameter (0D) model of the remaining cardiovascular portions including the heart, distal arteries, microcirculations, veins and the pulmonary circulation, thereby forming a closed-loop representation of the global cardiovascular system. The gas exchange and transport part mainly accounted for the intake, transport and consumption of oxygen in the cardiovascular system, and was composed of sub-models representing gas exchanges in the lung, microcirculation, and membrane oxygenator and the transport of gases in the cardiovascular system. It is noted that herein we assumed that gas exchange and transport are affected by local hemodynamic conditions, but do not affect the state of blood flow in turn. For more details on the modeling of the native cardiovascular system, we refer interested readers to our previous studies (Liang et al., 2009a; Liang et al., 2009b), and hereinafter we focus on introducing the modeling methods for ECMO, gas exchange and transport, as well as associated numerical schemes.

### 2.1 Modeling of ECMO and its coupling to the cardiovascular system

**FIGURE 2:**
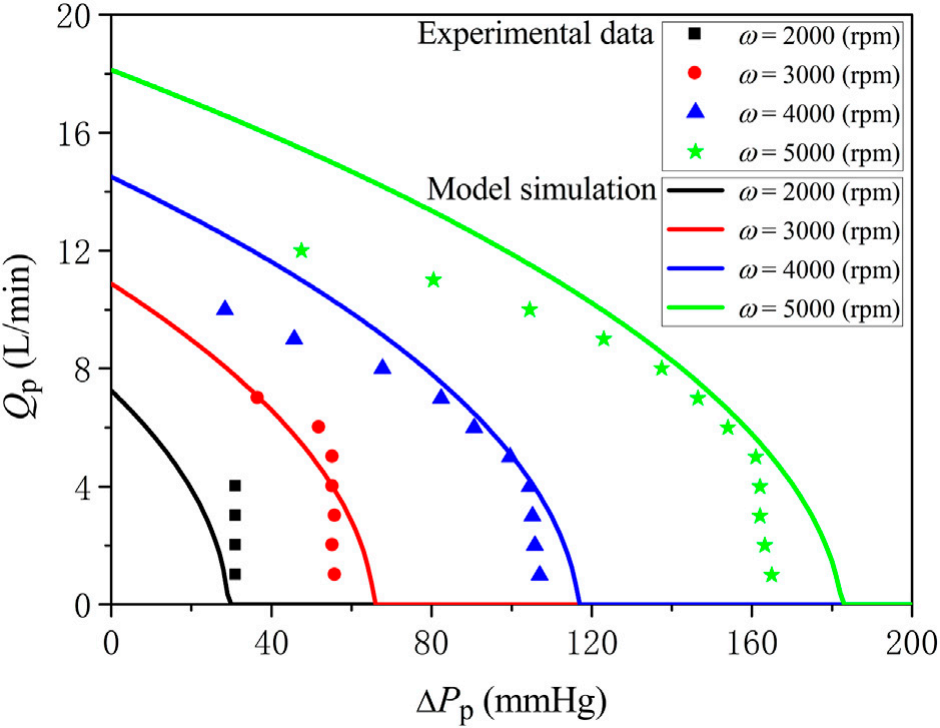
Comparison of model-simulated trans-pump pressure gradient-flow rate curves at various pump rotation speeds with experimental data.

The ECMO system is comprised mainly by the inflow cannula (also called drainage cannula), outflow cannula, blood pump, and membrane lung (or membrane oxygenator). In this study, for purpose of simplicity, we adopted the lumped-parameter modeling method to represent the ECMO system (see Figure 1). Specifically, the inflow cannula and outflow cannula were each represented by a combination of resistance, compliance and inertance that account for the viscous friction and deformability of cannula, and blood inertia, respectively. Blood pump was represented by a mathematical model established based on experimental data in a previous study (Shi and Korakianitis, 2018), and the membrane lung was modeled by reference to a gas exchange model for the native lung (Albanese et al., 2016) (will be detailed later). The model of blood pump was expressed in form of the relationship between pump flow rate (*Q*_p_) and trans-pump pressure gradient (∆*P*_p_) and pump rotation speed (*ω*).

$$Q_{p}=\frac{-\frac{K_{B}}{K_{A}}∙\omega +\sqrt{{\left(\frac{K_{B}}{K_{A}}\right)}^{2}∙\omega^{2}-\frac{4}{K_{A}}\left(\omega^{2}∙K_{C}-∆P_{p}\right)}}{2},$$ (1)

where *K*_*A*_, *K*_*B*_, and *K*_*C*_ are constants determined by the properties of pump, which were herein assigned following a previous study (Shi and Korakianitis, 2018), i.e., *K*_*A*_ = −0.0018 mmHg ∙ s^2^/ml^2^, *K*_*B*_ = −1.2 × 10^−5^ mmHg ∙ s/mL ∙ rpm, and *K*_*C*_ = 7.3 × 10^−6^ mmHg/rpm^2^. Figure 2 shows the model-simulated relationships between ∆*P*_p_ and *Q*_p_ at various pump rotation speeds compared against the experimental data (Bourque et al., 2001). Under various working conditions of the pump, the mean error of model predictions for ∆*P*_p_ compared against experimental data was 5.02 mmHg, with a standard deviation of 10.5 mmHg, which is consistent with the results reported in (Shi and Korakianitis, 2018).

The ECMO model was coupled to the native cardiovascular system by connecting the inlet of the inflow cannula to the right atrium while the outlet of the outflow cannula to an artery (aortic arch in the case of central VA-ECMO, whereas right external iliac artery in the case of peripheral VA-ECMO). Gas exchange of blood flowing through the membrane lung was modeled in a similar way as the modeling of gas exchange in the native lung, and the transport of gases contained by the blood through the ECMO cannulas was governed by lumped-parameter models, both of which will be detailed later.

### 2.2 Modeling of gas exchanges in the native lung, membrane lung, and microcirculation

A comprehensive lumped-parameter model for gas exchange in the native lung has been proposed and validated in a previous study (Albanese et al., 2016), and was herein adopted with minimal modifications to represent pulmonary dysfunction. In brief, the model was built based on the mass conservation principle for oxygen (O_2_) and carbon dioxide (CO_2_). The model took the fractions of O_2_ ( $F_{i,O_{2}}$ ) and CO_2_ ( $F_{i,{CO}_{2}}$ ) in the inspired air, variables (e.g., dead-space, alveolar volume) computed by a lung mechanics model, and pulmonary arterial blood flow rate computed by a hemodynamic model as the inputs to compute the contents of O_2_ and CO_2_ in the pulmonary capillaries. The parameters of the model can be readily varied to represent various physiological or pathological conditions of the lung. For a more detailed description of model development and validation, interested readers are invited to refer to (Albanese et al., 2016). Given the main purpose of our study being to simulate the transport of O_2_ in the cardiovascular system, we herein represented the influence of pulmonary dysfunction on gas exchange in a simplified way by increasing the fraction of pulmonary shunt flow in the model. The membrane lung in the ECMO system works in a similar way as the native lung, to which the model of the native lung was applied following the methodology adopted in previous studies (Zanella et al., 2016; Joyce et al., 2018). With the model, the changes in the fraction of oxygen delivered to the oxygen blender can be readily simulated by varying $F_{i,O_{2}}$ .

Gas exchange within each individual tissue/organ was represented by a gas exchange model accounting for O_2_ consumption and CO_2_ production in the capillary bed. The model was established following the mass conservation principle for O_2_ and CO_2_, and expressed in form of the rates of time variations in O_2_ and CO_2_ contents determined by the amounts of gases transported from the upstream vessels and the rates of local O_2_ consumption and CO_2_ production.

$$V_{j}∙\frac{dC_{j,O_{2}}}{dt}=Q_{j}∙\left(C_{j-1,O_{2}}-C_{j,O_{2}}\right)-M_{j,O_{2}},$$ (2)

$$V_{j}∙\frac{dC_{j,{CO}_{2}}}{dt}=Q_{j}∙\left(C_{j-1,{CO}_{2}}-C_{j,{CO}_{2}}\right)+M_{j,{CO}_{2}},$$ (3)

where $V_{j},C_{j,O_{2}},C_{j,{CO}_{2}},Q_{j},M_{j,O_{2}},and\;M_{j,{CO}_{2}}$ represent blood volume, content of O_2_, content of CO_2_, blood flow rate, the rate of O_2_ consumption, and the rate of CO_2_ production in a capillary bed, respectively. Subscript ‘*j*’ denotes the ‘capillary segment’ along a vascular subsystem, accordingly, subscript ‘*j*-1’ represents the upstream vascular segment (arteriolar segment in our model). The values of $M_{j,O_{2}}\;and\;M_{j,{CO}_{2}}$ were taken from a previous study (Albanese et al., 2016) to represent the general metabolic conditions of the human body. It is noted that given the higher anatomical complexity of our model, the values of $M_{j,O_{2}}\;and\;M_{j,{CO}_{2}}$ in some organs/tissues could not be derived directly from the study (Albanese et al., 2016), which were herein estimated based on the blood flow rates by assuming that $M_{j,O_{2}}\;and\;M_{j,{CO}_{2}}$ are proportional to blood flow rate for organs/tissues belonging to the same category of body composition (e.g., splanchna, skeletal muscle, and brain).

### 2.3 Modeling of gas transport in the cardiopulmonary system

#### 2.3.1 Governing equations

Transport of gases in the entire cardiopulmonary system followed the mass conservation principle, but had different mathematical expressions depending on the geometrical scales of the hemodynamic models corresponding to specific cardiopulmonary portions. For distal arteries, arterioles, venules, veins and ECMO cannulas in which hemodynamic variables are represented by lumped-parameter (0D) models, the transport of O_2_ and CO_2_ was described by ordinary differential equations expressed similarly to Eqs 2, 3, but with the source terms being set to zero. For the transport of O_2_ and CO_2_ in large arteries to which the 1D hemodynamic modeling method was applied, partial differential equations were employed to describe the spatio-temporal variations in gas content caused by pulsatile blood flow, molecular diffusion and metabolic gas consumption/production. The blood carries O_2_ in two forms, i.e., dissolved O_2_ in plasma [which determines the partial pressure of oxygen ( $P_{O_{2}}$ )] and that bound to hemoglobin (which is related to oxygen saturation), and accordingly two equations are required to characterize the transport of O_2_, which can be further combined to yield one nonlinear transport equation, that is numerically solvable but computationally intensive (Moore and Ethier, 1997). This problem can be simplified by introducing the following assumptions: 1) oxygen bounded to hemoglobin has negligible effect on $P_{O_{2}}$ , and 2) oxygen content can be estimated from $P_{O_{2}}$ (Spencer et al., 2006). With the assumptions and further assuming that the partial pressure of oxygen distributes uniformly in the cross section of artery, we were able to formulate the transport of O_2_ in an artery by reference to the 1D formation for mass transport in tubes (Marbach and Alim, 2019; Wu et al., 2022) as

$$\frac{\partial P_{O_{2}}}{\partial t}+u\frac{\partial P_{O_{2}}}{\partial x}=D_{O_{2}}\frac{\partial^{2}P_{O_{2}}}{\partial x^{2}}-\frac{M_{O_{2}}}{\alpha }$$ (4)

Similarly, the transport of CO_2_ was governed by

$$\frac{\partial P_{CO_{2}}}{\partial t}+u\frac{\partial P_{{CO}_{2}}}{\partial x}=D_{{CO}_{2}}\frac{\partial^{2}P_{CO_{2}}}{\partial x^{2}}+\frac{M_{CO_{2}}}{\beta }$$ (5)

where *t* is time,*x* the coordinate along the axis of artery, and *u* the cross-sectional mean blood flow velocity. $P_{O_{2}}$ and $P_{CO_{2}}$ stand for the cross-sectional mean partial pressures of O_2_ and CO_2_, respectively. $D_{O_{2}}$ and $D_{{CO}_{2}}$ represent the diffusion coefficients of O_2_ and CO_2_ in blood, respectively, *α* and *β* refer respectively to the solubility coefficients of O_2_ and CO_2_ in plasma. $M_{O_{2}}$ and $M_{CO_{2}}$ denote the rates of oxygen consumption and CO_2_ production per unit area of arterial wall, respectively. The coefficients were assigned following previous studies (Gros and Moll, 1971; Ji et al., 2010; Albanese et al., 2016), i.e., $D_{O_{2}}$ = 1.2 × 10^−9^ m^2^/s, $D_{{CO}_{2}}$ = 1.1 × 10^−9^ m^2^/s, α = 2.5 × 10^−5^ mL O_2_/ml blood/mmHg, β = 0.03 mL CO_2_/ml blood/mmHg, and $M_{O_{2}}$ = 2.1 × 10^−5^ mL O_2_/ml blood ∙ s. It is noted that the ratio of $M_{CO_{2}}$ to $M_{O_{2}}$ was fixed at 0.84 (Albanese et al., 2016).

Given $P_{O_{2}}$ and $P_{{CO}_{2}}$ , the contents of oxygen ( $C_{O_{2}}$ ) and carbon dioxide ( $C_{{CO}_{2}}$ ) can be calculated using empirical models proposed by Spencer et al. (2006).

$$C_{O_{2}}=C_{sat,O_{2}}∙\frac{{\left(X_{O_{2}}\right)}^{\frac{1}{h_{1}}}}{1+{\left(X_{O_{2}}\right)}^{\frac{1}{h_{1}}}},with\;X_{O_{2}}=P_{O_{2}}∙\frac{1+\beta_{1}∙P_{CO_{2}}}{K_{1}∙\left(1+\alpha_{1}∙P_{CO_{2}}\right)},$$ (6)

$$C_{{CO}_{2}}=C_{sat,{CO}_{2}}∙\frac{{\left(X_{{CO}_{2}}\right)}^{\frac{1}{h_{2}}}}{1+{\left(X_{{CO}_{2}}\right)}^{\frac{1}{h_{2}}}},with\;X_{{CO}_{2}}=P_{{CO}_{2}}∙\frac{1+\beta_{2}∙P_{O_{2}}}{K_{2}∙\left(1+\alpha_{2}∙P_{O_{2}}\right)}\;.$$ (7)

As will be described later, the calculated $C_{O_{2}}$ and $C_{{CO}_{2}}$ on the 1D model side were used to formulate the interface conditions between the 1D model and 0D model.

Given the partial pressure and content of oxygen, oxygen saturation ( $S_{O_{2}}$ ) can be further calculated using an empirical equation (Albanese et al., 2016).

$$S_{a,O_{2}}=\frac{C_{a,O_{2}}∙100-P_{a,O_{2}}∙0.003}{Hgb∙1.34}\;.$$ (8)

#### 2.3.2 Boundary conditions

The aforementioned 1D governing equations for gas transport are constructed for each individual artery, which must be supported by certain boundary conditions so that gas transport in an arterial tree composed of multiple arteries and its coupling to gas transport in peripheral tissues/organs (represented by a 0D model) can be solved. Boundary conditions are present at the bifurcations of arteries within the arterial tree (i.e., bifurcation conditions) and at the aortic root and the distal ends of peripheral arteries where the 1D and 0D models are coupled together (i.e., 0-1D interface conditions).

At the bifurcations, continuity of gas partial pressure was imposed (Ji et al., 2010).

$$P_{{O_{2}}_{p}}=P_{{O_{2}}_{a}}=P_{{O_{2}}_{b}},P_{{{CO}_{2}}_{p}}=P_{{{CO}_{2}}_{a}}=P_{{{CO}_{2}}_{b}},$$ (9)

where subscript ‘p’ denotes the parent artery, while ‘a’ and ‘b’ the daughter arteries.

At the aortic root and distal ends of peripheral arteries where the 0-1D interfaces reside, two sets of conditions were prescribed. One was applied to the arterial side by assuming that the diffusion of gas partial pressure is zero.

$$D_{O_{2}}\frac{\partial^{2}P_{O_{2}}}{\partial x^{2}}=0,D_{{CO}_{2}}\frac{\partial^{2}P_{CO_{2}}}{\partial x^{2}}=0$$ (10)

The other one was imposed to guarantee the mass conservation of gases flowing through each 0-1D interface.

$$C_{{O_{2}}_{1D}}Q_{1D}=C_{{O_{2}}_{0D}}Q_{0D},C_{{{CO}_{2}}_{1D}}Q_{1D}=C_{{{CO}_{2}}_{0D}}Q_{0D},$$ (11)

where ‘*C*’ represents the content of O_2_ or CO_2_, with the subscript ‘1D’ denoting the aortic root or the distal end of a peripheral artery represented by the 1D model, while ‘0D’ denoting the aortic valve or the proximal end of the distal vascular subsystem represented by the 0D model. ‘*Q*’ is the volumetric blood flow rate, which when multiplied by ‘*C*’ stands for the mass flow rate of gas.

### 2.4 Numerical methods

Numerical methods used to solve the governing equations of the hemodynamic models were the same as those developed in our previous studies (Liang et al., 2009a; Liang et al., 2009b). In this study, we assumed that the intake, consumption, and transport of gases do not affect the state of blood flow so that the governing equations for gas transport can be solved separately. Specifically, the 0D ordinary differential equations were solved using the fourth-order Runge-Kutta method, whereas the 1D partial differential equations were solved using a finite difference method where the time derivative term, convective term and diffusive term are discretized with the explicit first-order Euler, first-order upwind and second-order central differencing schemes, respectively. It is noted that the adoption of first-order numerical schemes for some terms helps to reduce the complexity of programming, but would have little influence on the reliability of numerical solution since we set the numerical time step (∆*t*) and grid size (∆*x*) to be sufficiently small (∆*t* = 2.5 × 10^−5^ s, ∆*x* = 1 × 10^−3^ m). At each time step, the values of hemodynamic variables (e.g., flow velocity, volumetric flow rate, blood pressure) involved in the governing equations of gas exchange and transport were taken directly from the converged solution of the hemodynamic model obtained in prior by running the hemodynamic simulation for a sufficiently long time (usually 10 to 15 cardiac cycles). In order to solve gas variables at arterial bifurcations, we assumed that the contributions of the diffusion and source terms are negligible so that a ‘ghost point’ method (Liang et al., 2009a) can be applied to solve the simplified gas transport equation using the information contained by the last two grids of the parent artery, and the solution was then used to update gas variables in the other two arteries using Eq. 9. Similar assumptions and numerical schemes were adopted when solving gas variables at the 0-1D interfaces.

### 2.5 Model verification

**FIGURE 3:**
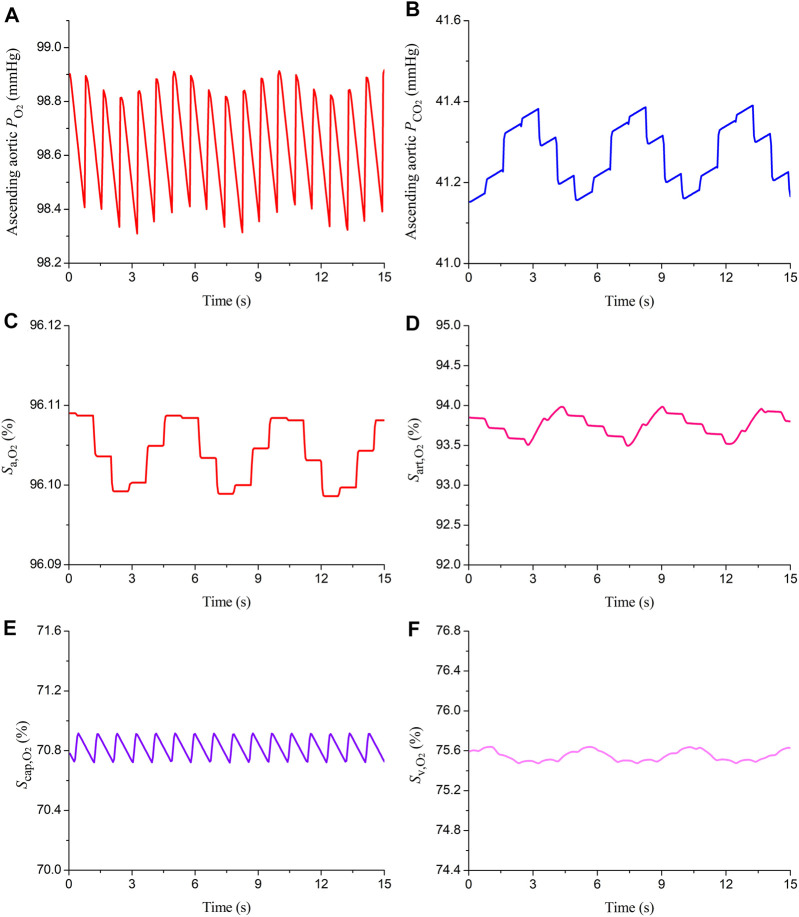
Simulated time courses of partial pressures of oxygen and carbon dioxide ( $P_{O_{2}}$ ; $P_{{CO}_{2}}$ ) and oxygen saturation ( $S_{a,O_{2}}$ ) in the ascending aorta **(A–C)**, oxygen saturations in the arterioles and capillary bed in the spleen [denoted respectively by S_art,O2_ and S_cap,O2_ in **(D,E)**], and mixed venous oxygen saturation (S_v,O2_) **(F)** under normal physiological conditions. The data are presented for 15 s which cover three respiratory cycles.

The model was firstly applied to simulate blood flow and gas transport in the cardiovascular system of a healthy young subject by assigning model parameters based on the data reported in the literature (Liang et al., 2009a; Liang et al., 2009b; Albanese et al., 2016). The values of major model parameters determining blood flow rates and metabolic consumptions of oxygen in various organs/tissues are listed in Table 1. Table 2 shows the model-simulated hemodynamic and gas variables compared against physiological data (Comroe, 1977; Heldt et al., 2002; Arthurs and Sudhakar, 2005; Edwards Lifesciences Corporation, 2014). All the model-simulated results fell in the physiological ranges. Figure 3 further shows the model-simulated time courses of the partial pressures of O_2_ and CO_2_ in the ascending aorta, as well as blood oxygen saturations in the ascending aorta, the arterioles and capillary bed in the spleen, and mixed venous oxygen saturation. It was observed that the values of the gas variables changed mildly over time in two periodic patterns, one was in sync with the pulsation of blood flow with a period of 0.833 s, while the other one was in line with the rhythm of respiration (one cycle is 5 s), reflecting the influences of blood flow and respiration on gas exchange and transport. It is interesting to remark that the slightly lower value of oxygen saturation in the capillary bed of the spleen than the mixed venous oxygen saturation is caused by the relatively high rate of metabolic consumption of oxygen in the spleen compared to many other organs/tissues (see Table 1). Overall, these results indicate that our model can reasonably delineate the general hemodynamic characteristics and gas transport behavior in the human body.

**TABLE 1:** Values of the total resistance (*R*_t_), compliance (*C*_t_), and inertance (*L*_t_) of the vascular system distal to each peripheral artery and the rates of O_2_ consumption ( $M_{O_{2}}$ ) and CO_2_ production ( $M_{CO_{2}}$ ) in the organ/tissue perfused by the artery.

Peripheral arteries	*R*_t_ (mmHg·s·ml^-1^)	*C*_t_ (ml·mmHg^-1^)	*L*_t_ (mmHg·s^2^·ml^-1^)	$M_{O_{2}}$ / $M_{CO_{2}}$ (ml·min^-1^)	Organ/Tissue
R./L. radius	51.462	1.259 × 10^−3^	0.110	3.314/2.783	Upper limbs
R./L. interosseous	831.945	7.9 × 10^−5^	0.441	0.213/0.179	
R./L. ulnar II	51.687	1.259 × 10^−3^	0.110	3.393/2.850	
R./L. int.iliac	54.615	1.197 × 10^−3^	0.113	3.277/2.752	Lower limbs
R./L. deep femoral	32.764	1.991 × 10^−3^	0.088	5.381/4.520	
R./L. post.tibial	72.804	8.97 × 10^−4^	0.131	2.353/1.976	
R./L. ant.tibial	32.820	1.991 × 10^−3^	0.088	5.297/4.450	
R./L. PCA II	107.973	6.0 × 10^−4^	0.160	5.732/4.815	Brain
R./L. MCA	58.374	1.113 × 10^−3^	0.117	11.989/10.071	
R./L. ACA II	82.768	7.84 × 10^−4^	0.140	6.030/5.065	
R./L. sup. thy. asc. ph. lyng. fac. occ.	294.408	2.21 × 10^−4^	0.263	0.542/0.455	
R./L. maxillary	245.340	2.65 × 10^−4^	0.240	0.648/0.545	
R./L. superf. temp. fron. bran.	245.340	2.65 × 10^−4^	0.240	0.691/0.581	
R./L. superf. temp. pari. bran.	245.340	2.65 × 10^−4^	0.240	0.691/0.580	
Splenic	53.057	1.229 × 10^−3^	0.112	9.418/7.911	Spleen
Gastric	22.733	2.865 × 10^−3^	0.073	21.939/18.429	Stomach
Sup. mesenteric	9.136	7.148 × 10^−3^	0.046	55.249/46.409	Intestines
Inf. mesenteric	67.780	9.66 × 10^−4^	0.126	7.444/6.253	
Hepatic	35.763	1.863 × 10^−3^	0.092	14.369/12.070	Liver
R./L. renal	11.070	5.883 × 10^−3^	0.051	5.263/4.421	Kidney
Intercostal	13.643	4.782 × 10^−3^	0.057	4.157/3.492	Chest tissues

**TABLE 2:** Comparisons of model-simulated hemodynamic and gas variables with physiological data.

Variable	Model simulation	Physiological data
Mean arterial pressure (mmHg)	97.932	70–105 (Edwards Lifesciences Corporation, 2014)
Mean central venous pressure (mmHg)	8.281	6–9 (Heldt et al., 2002)
Left-ventricle systolic Pressure (mmHg)	124.192	90–140 (Heldt et al., 2002)
Left-ventricle end-diastolic pressure (mmHg)	4.536	4–12 (Heldt et al., 2002)
Right-ventricle systolic pressure (mmHg)	24.867	15–28 (Heldt et al., 2002)
Right-ventricle end-diastolic pressure (mmHg)	1.708	0–8 (Heldt et al., 2002)
Arterial $C_{O_{2}}$ (mmol/L)	8.726	8.748 (Comroe, 1977)
Arterial $C_{{CO}_{2}}$ (mmol/L)	21.432	21.348 (Comroe, 1977)
Mixed venous $C_{O_{2}}$ (mmol/L)	6.610	6.652 (Edwards Lifesciences Corporation, 2014)
Mixed venous $C_{{CO}_{2}}$ (mmol/L)	23.329	23.505 (Arthurs and Sudhakar, 2005)
Arterial oxygen saturation (%)	96.140	95–100 (Edwards Lifesciences Corporation, 2014)
Mixed venous saturation (%)	75.757	60–80 (Edwards Lifesciences Corporation, 2014)

### 2.6 Setup of numerical experiments

The general indications for VA-ECMO support are systemic hypoxia caused by severe cardiac failure or concomitant cardiac and pulmonary (or respiratory) failure (King et al., 2017; Lorusso et al., 2021), therefore, our numerical experiments were designed to investigate and compare the effects of central and peripheral VA-ECMO supports on hemodynamic characteristics and gas transport in the cardiovascular system under pathological conditions characterized by isolated cardiac failure or cardiopulmonary failure.

The classifications of cardiac failure and pulmonary failure have been well defined in the clinical field. For the left ventricle (LV), systolic failure is generally defined as an ejection fraction (EF) of <40% (Lam and Solomon, 2021), whereas pulmonary (or respiratory) failure is diagnosed when the arterial oxygen partial pressure is less than 60 mmHg, an indicator of severe arterial hypoxemia (Lamba et al., 2016). Our model-based numerical tests showed that the simulated left ventricular EF decreased to lower than 40% when the peak elastance of the LV [a parameter representing myocardial contractility in the model (Liang et al., 2009a)] was reduced to <36% of the normal value, and that the arterial oxygen partial pressure decreased to lower than 60 mmHg when the fraction of pulmonary shunt flow (FPSF) in the lung gas exchange model was increased over 62%. Based on the results of numerical tests, we adjusted the model parameters to simulate various pathological conditions characterized by isolated severe cardiac failure or severe cardiopulmonary failure (i.e., cardiac failure accompanied by pulmonary dysfunction). Herein, four pathological conditions were simulated, namely, ‘Cardiac Failure I’ and ‘Cardiac Failure II’ represented by reducing the peak elastances of the left and right ventricles relative to the normal value by 80% and 90%, respectively, ‘Cardiopulmonary Failure I’ represented by reducing the peak elastances of the left and right ventricles by 80% and increasing FPSF to 80%, and ‘Cardiopulmonary Failure II’ represented by reducing the peak elastances of the left and right ventricles by 90% and increasing FPSF to 90%. It is noted that the peak elastances of the left and right ventricles were decreased simultaneously to represent biventricular failure, a pathological condition expected to especially benefit from VA-ECMO support (King et al., 2017; Lorusso et al., 2021). The values of model parameters corresponding to the four pathological conditions are summarized in Table 3.

**TABLE 3:** Assigned parameter values for representing four pathological conditions. Note that the parameter values in the normal condition are also provided as a reference.

Model parameters	Normal condition	Cardiac failure I	Cardiac failure II	Cardiopulmonary failure I	Cardiopulmonary failure II
Peak elastance of LV (mmHg·ml^-1^)	2.87	0.574	0.287	0.574	0.287
Peak elastance of RV (mmHg·ml^-1^)	0.52	0.104	0.052	0.104	0.052
Fraction of pulmonary shunt flow (%)	1.7	1.7	1.7	80	90

Numerical simulations were performed for each pathological condition to compare the central and peripheral VA-ECMO modalities with respect to their hemodynamic impact and effectiveness of oxygen supply to important end-organs/tissues. In addition, numerical simulations with different ECMO pump rotation speeds and fractions of oxygen delivered to the oxygen blender were also carried out to analyze the sensitivities of hemodynamic and gas variables of interest to the operating parameters of ECMO. The outflow cannula of ECMO was connected to the aortic arch in the case of central VA-ECMO, and to the right external iliac artery in the case of peripheral VA-ECMO. In addition, in all the simulations, the resting heart rate was fixed at 72 bpm, and the hemodynamic effects of autonomous respiration were introduced via cyclically varying intrathoracic pressure from −5.8 mmHg to −3.3 mmHg over a period of 5 s.

The metabolic activities of organs/tissues and the associated demands for oxygen may change under ischemic or hypoxemic conditions. Previous animal experimental studies (Edelstone et al., 1983; Szabo et al., 1987) demonstrated that oxygen uptake by splanchna could remain stable despite the variations of oxygen supply over a wide range until oxygen supply was reduced below a critically low threshold. For purpose of simplicity, we assumed that the metabolic demands for oxygen of organs/tissues do not change with the pathological conditions of the heart or lung. Accordingly, the values of the metabolic terms in Eqs 2, 3 were fixed at the normal physiological state (given in Table 1) throughout our study. The initial conditions of hemodynamic and gas variables were estimated by reference to physiological values, and were applied to all the models irrespective of the differences in simulated pathological conditions. We stress that artifacts introduced in the assignment of initial conditions will not alter the final numerical solution since the closed-loop configuration of the model guarantees that a global equilibrium of gas intake, production and consumption can be reached as long as the numerical simulation converges sufficiently.

## 3 Results

### 3.1 Characteristics of hemodynamics and gas transport in the arterial system under central and peripheral VA-ECMO supports

**FIGURE 4:**
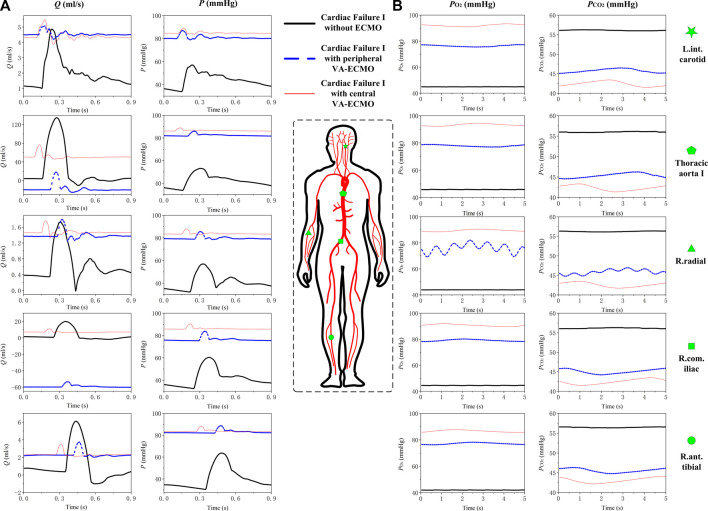
Simulated blood pressure/flow waveforms **(A)** and time courses of $P_{O_{2}}$ ; $P_{{CO}_{2}}$ **(B)** in five representative arteries under the ‘Cardiac Failure I’ condition without ECMO support or with peripheral or central VA-ECMO support. The rotation speed of ECMO pump is set at 5,400 rpm. Abbreviations: L. int. carotid, left internal carotid artery; R. radial, right radial artery; R. com. iliac, right common iliac artery; R. anti. tibial, right anterior tibial artery.

Numerical simulations were firstly performed for the ‘Cardiac Failure I’ condition to investigate how central and peripheral VA-ECMO supports differ with respect to their effects on hemodynamics and gas transport in the arterial system. To facilitate comparison, the same ECMO operating parameters (i.e., *ω* = 5,400 rpm and $F_{i,O_{2}}$ = 100%) have been assigned to the two VA-ECMO modalities. Figure 4 displays the simulated blood pressure and flow waveforms and time courses of the partial pressures of oxygen ( $P_{O_{2}}$ ) and carbon dioxide ( $P_{{CO}_{2}}$ ) in five representative arteries (i.e., left internal carotid artery, thoracic aorta, right radial artery, right common iliac artery, and right anterior tibial artery) without ECMO support and with central and peripheral VA-ECMO supports, respectively. Both ECMO supports remarkably raised the mean blood pressure in all the arteries, but led to an evident attenuation of pressure pulsation. The weakening of pulsation was observed for blood flow as well, but the changes in flow waveform differed evidently among arteries and exhibited marked differences between the two ECMO modalities. For instance, blood flow rate increased evidently in all peripheral arteries that perfuse certain organs/tissues (e.g., left internal carotid artery, right radial artery, and right anterior tibial artery) irrespective of the modality of ECMO, whereas the changes of blood flow waveforms in the thoracic aorta and the right common iliac artery depended strongly on ECMO modality. Specifically, the central VA-ECMO induced a marked increase of blood flow in both the thoracic aorta and the right common iliac artery; by contrast, the peripheral VA-ECMO reversed the direction of blood flows in the thoracic aorta and right common iliac artery. In addition, the increase of arterial blood pressure was slightly larger with central VA-ECMO support than with peripheral VA-ECMO support, although the operating parameters of the two ECMO systems were set to be the same.

With respect to the transport of gases in the arterial system, it was observed that $P_{O_{2}}$ was much lower while $P_{{CO}_{2}}$ much higher than the normal physiological values (see Table 2) in the absence of ECMO assistance. In addition, $P_{O_{2}}$ decreased slightly from the aorta toward the peripheral arteries (e.g., the R. ant. tibial artery) as a consequence of the mild consumption of oxygen by arterial wall. Once VA-ECMO support was introduced, $P_{O_{2}}$ rose remarkably in all the arteries, whereas $P_{{CO}_{2}}$ decreased evidently, which demonstrated the major role of ECMO in relieving hypoxemia caused by cardiac failure. The differences in $P_{O_{2}}$ or $P_{{CO}_{2}}$ among arteries were observable but relatively small in magnitude. Moreover; $P_{O_{2}}$ was overall higher with central VA-ECMO support than that with peripheral VA-ECMO support. Differently from blood flow; $P_{O_{2}}$ ; $P_{{CO}_{2}}$ in most arteries did not change intensively with time in each cardiac cycle, only exhibiting moderate periodic variations at interval of the respiration duration (5s). An exception is that $P_{O_{2}}$ and $P_{{CO}_{2}}$ in the right radial artery oscillated considerably. Given the oscillation period being equal to the cardiac cycle, we speculate that the dynamic interaction between the pulsatile forward flow sourced from the heart and the backward flow from the ECMO may be a major causative factor, although further studies capable of separately tracing the distribution of oxygen components sourced from the lung and the ECMO would be required to more clearly reveal the underlying mechanisms.

### 3.2 Sensitivities of aortic oxygen saturation and mixed venous oxygen saturation to the operating parameters of ECMO

**FIGURE 5:**
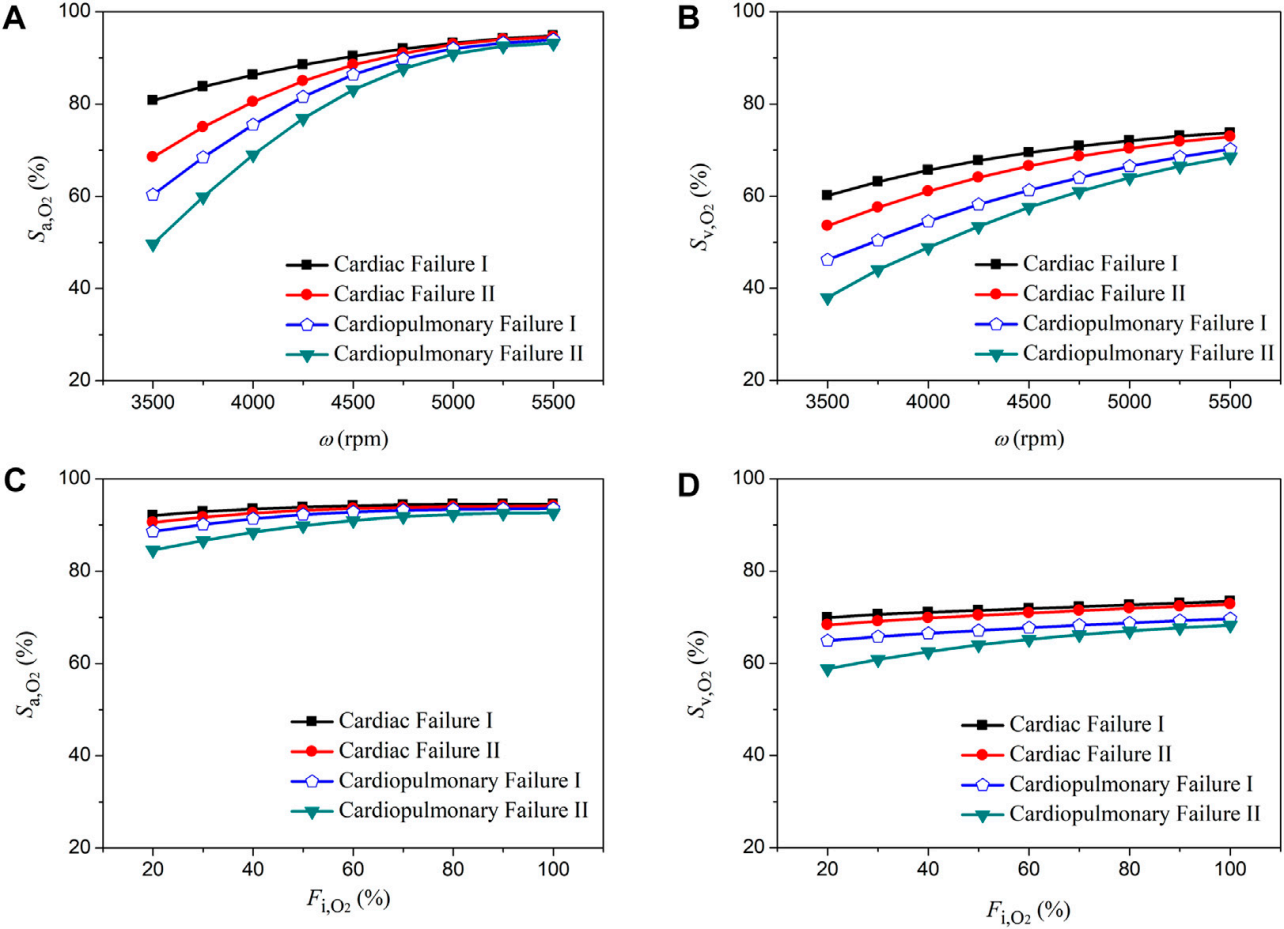
Changes in aortic oxygen saturation ( $S_{a,O_{2}}$ ) and mixed venous oxygen saturation ( $S_{v,O_{2}}$ ) with the increase of the rotation speed of ECMO pump (*ω*) from 3,500 rpm to 5,500 rpm **(A,B)** and with the increase of the fraction of oxygen ( $F_{i,O_{2}}$ ) delivered to the oxygen blender from 20% to 100% **(C,D)** under four pathological conditions with central VA-ECMO support.

**FIGURE 6:**
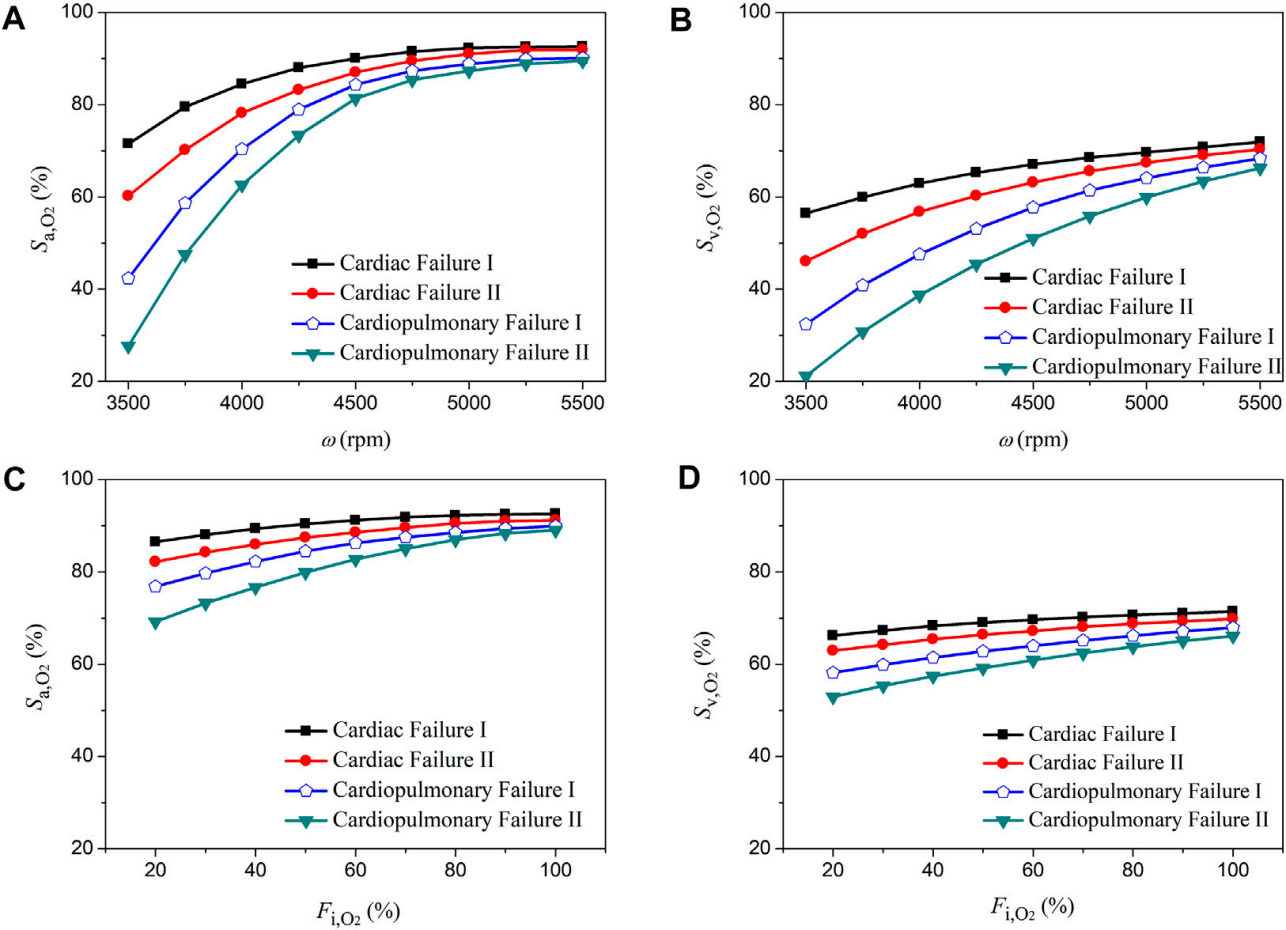
Changes in aortic oxygen saturation ( $S_{a,O_{2}}$ ) and mixed venous oxygen saturation ( $S_{v,O_{2}}$ ) with the increase of the rotation speed of ECMO pump (*ω*) from 3,500 rpm to 5,500 rpm **(A,B)** and with the increase of the fraction of oxygen ( $F_{i,O_{2}}$ ) delivered to the oxygen blender from 20% to 100% **(C,D)** under four pathological conditions with peripheral VA-ECMO support.

The rotation speed of pump (*ω*) and the fraction of oxygen ( $F_{i,O_{2}}$ ) delivered to the oxygen blender are two major ECMO parameters that are usually adjusted to meet patient-specific extracorporeal blood oxygenation demand in clinical settings (King et al., 2017; Lorusso et al., 2021). We performed numerical simulations to investigate how variations in *ω* (from 3,500 rpm to 5,500 rpm) and $F_{i,O_{2}}$ (from 20% to 100%) affect the oxygen saturation in the ascending aorta ( $S_{a,O_{2}}$ , which indicates the abundance of systemic oxygen supply) and the mixed venous oxygen saturation ( $S_{v,O_{2}}$ , which reflects the degree of balance between systemic oxygen supply and oxygen consumption) under various pathological conditions (i.e., ‘Cardiac Failure I’, ‘Cardiac Failure II’, ‘Cardiopulmonary Failure I’, and ‘Cardiopulmonary Failure II’). Figures 5, 6 show the simulated results for central VA-ECMO and peripheral VA-ECMO supports, respectively. Increasing *ω* (with $F_{i,O_{2}}$ being fixed at 100%) resulted in a marked increase in both $S_{a,O_{2}}$ and $S_{v,O_{2}}$ , especially when *ω* < 5,000 rpm. Relatively, the sensitivities of $S_{a,O_{2}}$ and $S_{v,O_{2}}$ to variations in $F_{i,O_{2}}$ (with *ω* being fixed at 5,400 rpm) were low, especially when $F_{i,O_{2}}$ was increased over 80%. Moreover, the sensitivities of $S_{a,O_{2}}$ ; $S_{v,O_{2}}$ to variations in *ω* or $F_{i,O_{2}}$ were augmented following the increase in the initial severity of hypoxemia (herein determined by the severity of cardiac failure or cardiopulmonary failure). For instance, under the condition of ‘Cardiopulmonary Failure II’, the sensitivities were highest. When the results for the two VA-ECMO modalities were compared, it was observed that the sensitivities of $S_{a,O_{2}}$ and $S_{v,O_{2}}$ to variations in *ω* were higher with peripheral VA-ECMO support than with central VA-ECMO support, especially when *ω* was low and the initial hypoxemia was severe.

### 3.3 Differences between oxygen saturations and partial pressures in the radial artery and those in the perfusion arteries of organs/tissues

The right radial artery is usually recommended as the site for monitoring blood oxygen saturation or partial pressure during ECMO support, however, whether blood oxygen indices monitored at this site could reliably indicate the level of blood oxygenation in other arteries remains unclear (Krishnan and Schmidt, 2019). In this study, we systemically compared the computed oxygen saturations and partial pressures in several representative arteries against those in the right radial artery. Herein, the left internal carotid artery, coronary artery, right renal artery, and right anterior tibial artery, which deliver blood to the brain, myocardium, kidney, and foot, respectively, were selected. Blood oxygen indices in these arteries and the right radial artery were derived from the computed results for the pathological condition ‘Cardiopulmonary Failure II’ during the aforementioned sensitivity analysis where *ω* and $F_{i,O_{2}}$ were each varied over a wide range.

**FIGURE 7:**
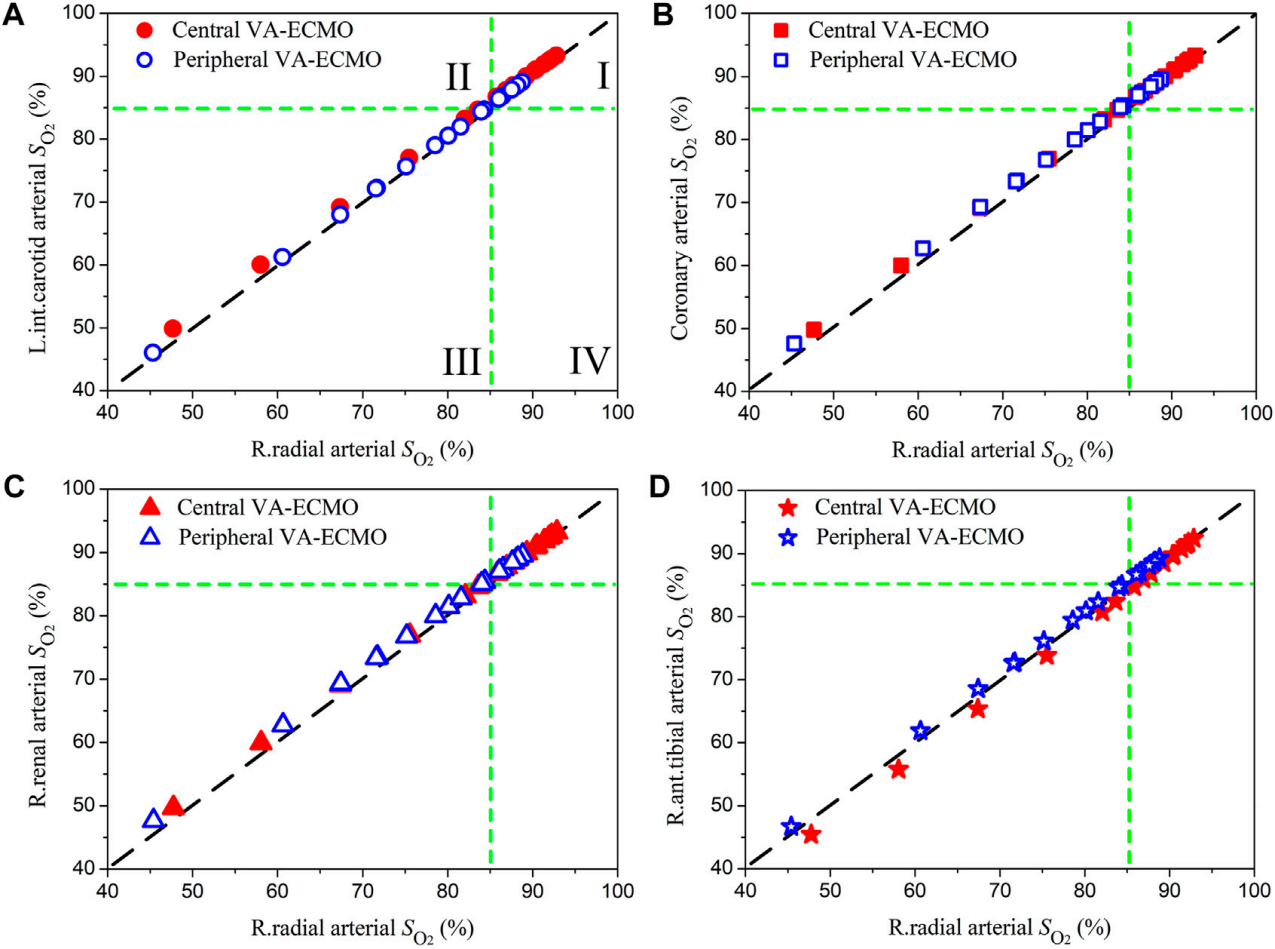
Comparisons of blood oxygen saturations ( $S_{O_{2}}$ ) in the left internal carotid artery **(A)**, coronary artery **(B)**, right renal artery **(C)**, and right anterior tibial artery **(D)** against those in the right radial artery. The data are plotted in form of scatter points, where the blank and filled icons indicate the computed results for peripheral and central VA-ECMO supports, respectively. In each panel, two lines are drawn at $S_{O_{2}}=$ 85% (the threshold for judging severe hypoxemia) to divide the panel into four quadrants from which missed diagnosis or misdiagnosis of hypoxemia in any arteries using $S_{O_{2}}$ in the radial artery can be clearly identified.

**FIGURE 8:**
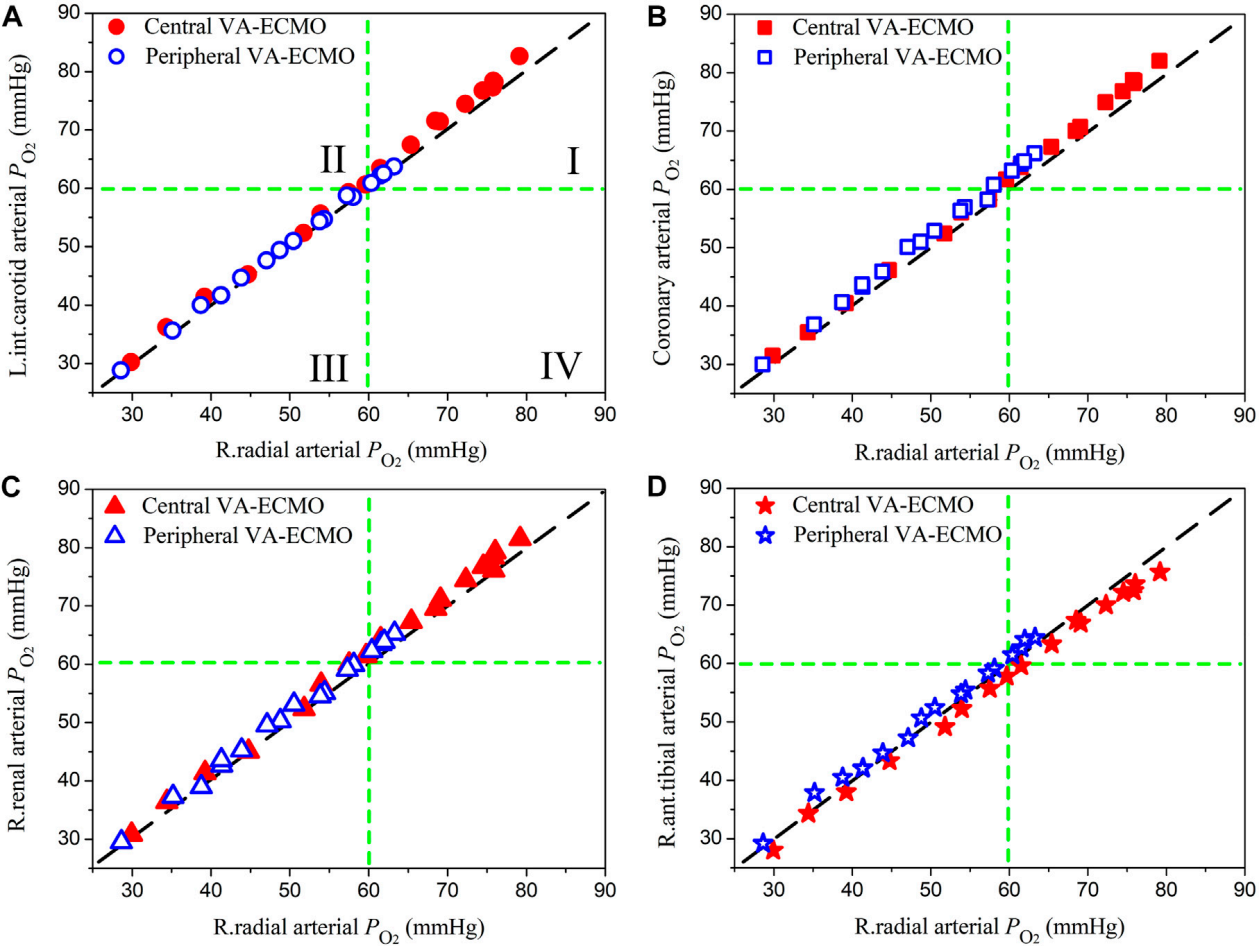
Comparisons of oxygen partial pressures ( $P_{O_{2}}$ ) in the left internal carotid artery **(A)**, coronary artery **(B)**, right renal artery **(C)**, and right anterior tibial artery **(D)** against those in the right radial artery. The methods for plotting the data and dividing each panel are similar to those used in Figure 7, except that the threshold of $P_{O_{2}}$ for judging severe hypoxemia is set at 60 mmHg.

Figures 7, 8 show the data of oxygen saturation ( $S_{O_{2}}$ ) and partial pressure ( $P_{O_{2}}$ ), respectively. In each panel of Figure 7, two lines are drawn at $S_{O_{2}}$ = 85% [the threshold of $S_{O_{2}}$ for judging severe hypoxemia (Tyler et al., 1985; Gift et al., 1995)] to divide the panel into four quadrants. Data points located in quadrant I and quadrant III indicate that $S_{O_{2}}$ monitored at the radial artery can correctly reflect the severity of hypoxemia in other arteries, whereas data points dropping in quadrant II and quadrant IV indicate that $S_{O_{2}}$ monitored at the radial artery overestimate and underestimate the severity of hypoxemia in other arteries, respectively. The data of $P_{O_{2}}$ were plotted in Figure 8 in a similar way where the threshold is set at 60 mmHg (Lamba et al., 2016). Ideally, all the data points should distribute along the line of *y* = *x* if the blood oxygen indices in the radial artery are strictly equal to those in other arteries, which was however not the case from the results presented in Figures 7, 8 where many data points deviate from the *y* = *x* line, although the deviations are overall small. The majority of data points were in quadrant I or quadrant III, and the very few data points in quadrant II or quadrant IV did not exhibit large differences between oxygen indices in the radial artery and those in other arteries, which implies that blood oxygen indices monitored at the radial artery are basically indicative of the status of blood oxygenation in other arteries. Comparing the data obtained for the two ECMO modalities, we did not observe marked differences in terms of the relationships between radial arterial oxygen indices and those in other arteries, although $S_{O_{2}}$ and $P_{O_{2}}$ were overall lower in the case of peripheral VA-ECMO than the case of central VA-ECMO. Similar phenomena were observed on the computed data for the other three pathological conditions (data not shown).

### 3.4 Indicators of left ventricular overloading

VA-ECMO, despite its beneficial role in providing support for both cardiac and pulmonary functions, carries a risk of causing overloading of the left ventricle (LV) due to increased aortic afterload and associated poor LV ejection (Lorusso et al., 2021). LV overloading may be accompanied by intermittent or even permanent failure of aortic valve opening in systole, a condition under which the risk of acute pulmonary edema or thrombosis in cardiac chambers may increase dramatically (Lorusso et al., 2021). In this study, we took the pathological condition ‘Cardiac Failure I’ supported by central VA-ECMO as an example to address how the risk and severity of LV overloading change with the rotation speed of ECMO pump, a major determinant of the amount of blood flowing through the ECMO system and the efficiency of systemic blood oxygen compensation as shown in Figures 5, 6. Following the clinical guidelines for judging and classifying LV overloading (Lorusso et al., 2021), the frequency of aortic valve opening, the end-diastolic volume of the LV (LVEDV), and central venous pressure (CVP) were selected as the major parameters for assessing the severity of LV overloading.

**FIGURE 9:**
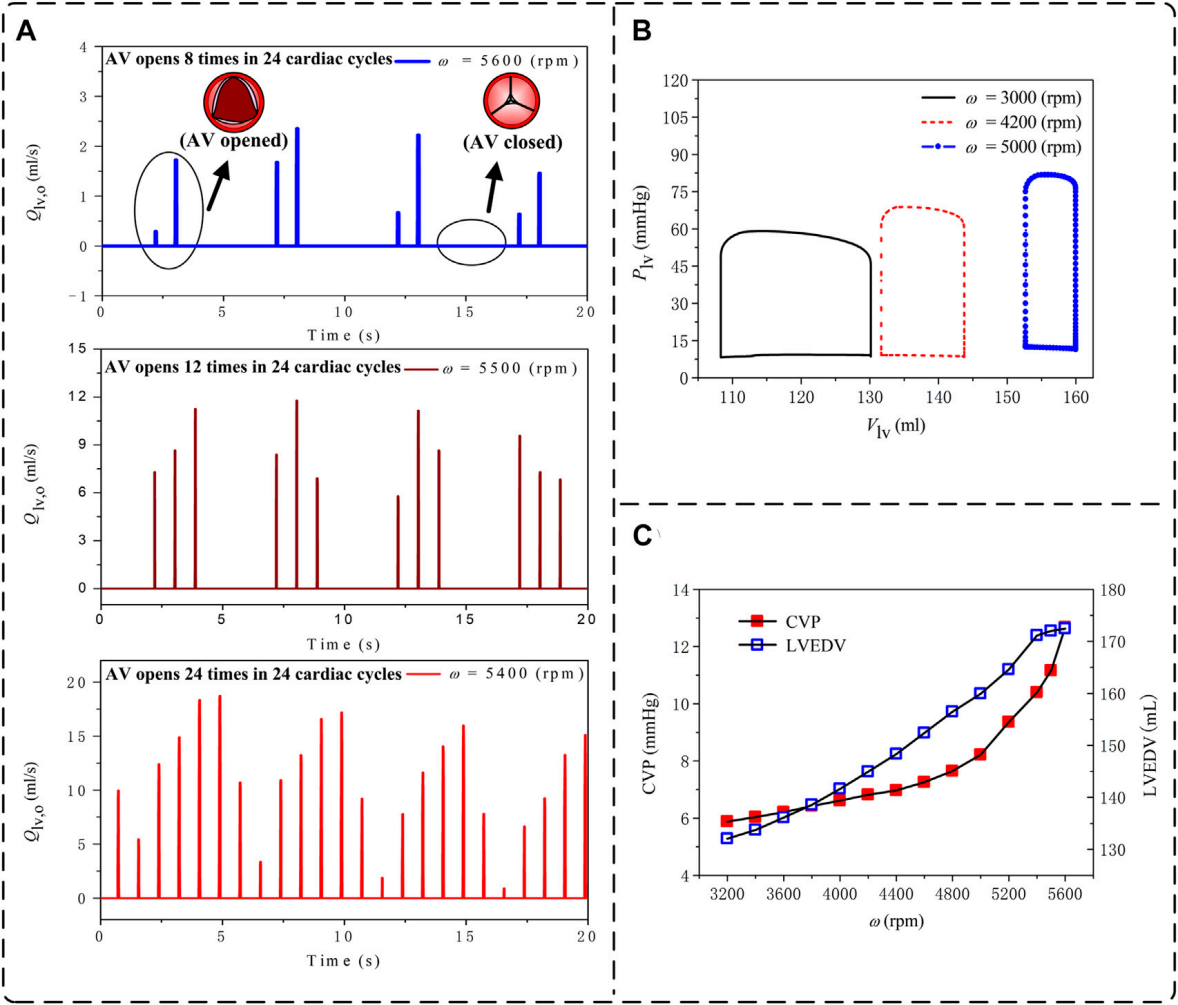
Model-simulated changes in hemodynamic variables used to assess the loading condition of the left ventricle (LV) with the increase of the pump rotation speed (*ω*) under the ‘Cardiac Failure I’ condition with central VA-ECMO support: **(A)** time courses of blood flow rates at the LV outlet over 24 cardiac cycles (*ω* = 5,600 rpm, 5,500 rpm, and 5,400 rpm); **(B)** pressure-volume loops of the LV (*ω* = 3,000 rpm, 4,200 rpm, and 5,000 rpm); and **(C)** central venous pressure (CVP) and LV end-diastolic volume (LVEDV). In **(A)**, opening of aortic valve (AV) is indicated by the appearance of a pulse flow in a cardiac cycle, whereas the persistent closed state of AV is indicated by the continuous zero flow rate during an entire cardiac cycle.

The three subfigures in Figure 9A display the simulated time courses of blood flow rate at the outlet of the LV during 24 cardiac cycles when the ECMO pump rotation speed (*ω*) was set at 5,600, 5,500 and 5,400 rpm, respectively. The frequency of aortic valve opening was one time every three cardiac cycles when *ω =* 5,600 rpm, which increased to one time every two cardiac cycles when *ω =* 5,500 rpm, and returned to the normal state (i.e., opened in every cardiac cycle) when *ω =* 5,400 rpm. In addition, the reduced frequency of aortic valve opening following the increase in *ω* was accompanied by markedly decreased magnitude and amplitude of blood flow through the aortic valve. From the LV pressure-volume (P-V) loops presented in Panel (B), it was observed that following the increase in *ω* the LV P-V loop exhibited a marked right and up shift along with a progressive reduction in stroke volume and elevation of both end-diastolic pressure and end-systolic pressure. To facilitate quantitative understanding, we plotted the model-simulated quantities of LVEDV and CVP against *ω* in Panel (C). Both LVEDV and CVP increased progressively with *ω*, although the specific patterns of increase differed between them. For instance, LVEDV increased with *ω* in a nearly linear manner [which is similar to the findings of a recent study (Colasanti et al., 2021)] unless *ω* was increased to higher than 5,400 rpm under which condition the opening of the aortic valve became abnormal, leading LVEDV to be disassociated from cardiac afterload and increase mildly with the increase of preload. In contrast, CVP increased moderately with *ω* as *ω* was low but more sharply following further increases in *ω*. By reference to the clinical criteria for grading the severity of LV overloading (Lorusso et al., 2021), the frequency of aortic valve opening and the level of CVP hit the borderlines of moderate severity (i.e., aortic valve opens every 3-4 cardiac cycles, CVP >12 mmHg) when *ω* was higher than 5,400 rpm.

## 4 Discussion

**FIGURE 10:**
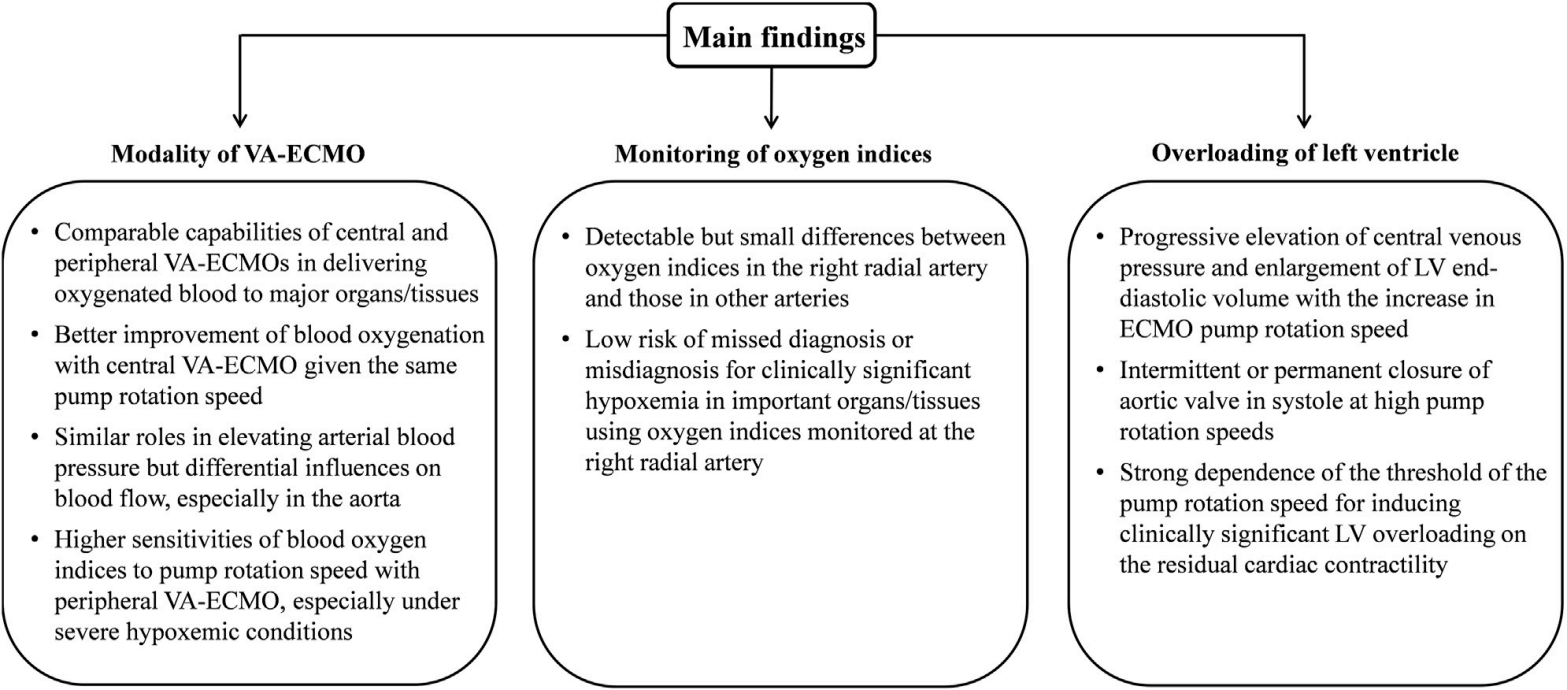
Diagrammatic description of the main findings.

The present study developed a 0-1D multi-scale model to quantitatively investigate the changes in hemodynamic variables and blood oxygen indices in response to the introduction of VA-ECMO support in the context of severe cardiac or cardiopulmonary failure. Methodologically, adopting the 1D modeling method for the arterial system in our study facilitated a detailed simulation of the interaction between blood flow sourced from the native heart and ECMO flow as well as its influence on gas transport in large arteries that are hard to be fully addressed by traditional 0D models (Zanella et al., 2016; Joyce et al., 2018). In comparison with existing open-loop 1D or 3D models of the arterial system (Stevens et al., 2018; Feiger et al., 2020; Khodaee et al., 2022), the closed-loop representation of the entire cardiopulmonary-ECMO system made our model better suited to address the combined effects on systemic hemodynamics and gas transport of the residual native blood circulation capacity, gas exchange/transport in the lung and microcirculations, and ECMO support. The main findings of our study are summarized in Figure 10 and described in detail as follows.

**FIGURE 11:**
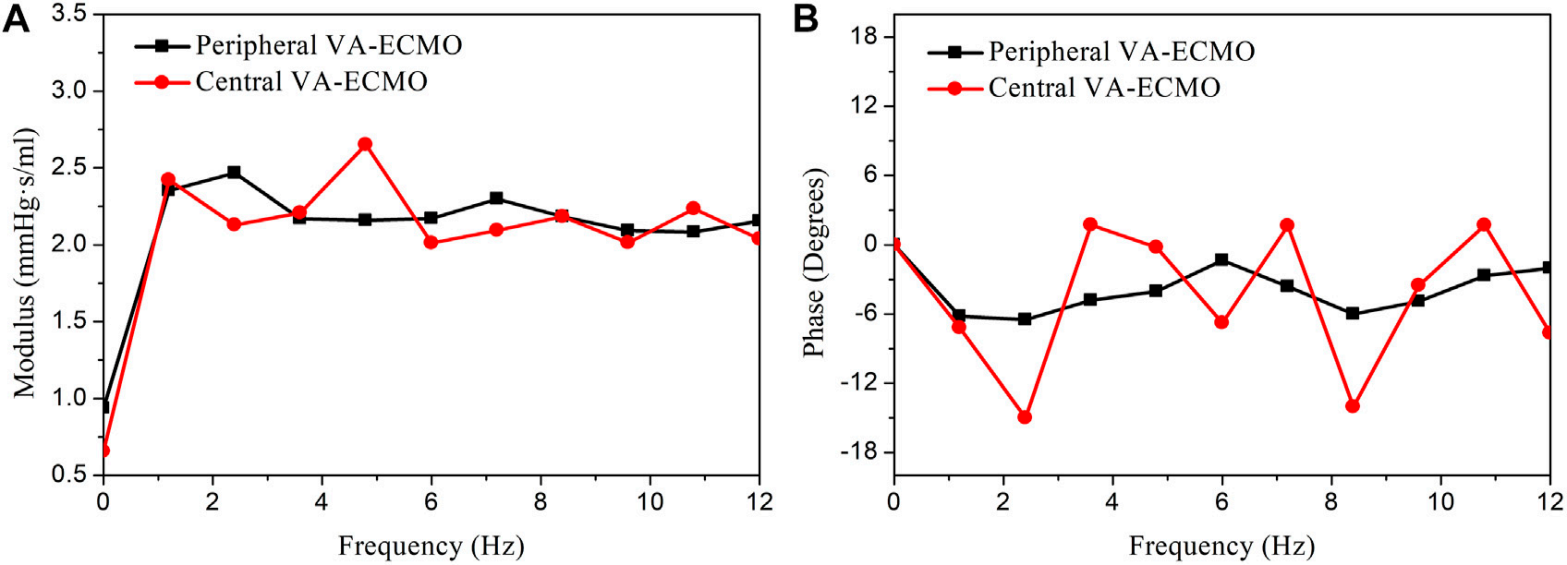
Comparison of the input impedances at the outflow cannula outlets of the peripheral and central VA-ECMO systems under the ‘Cardiac Failure I’ condition. The impedances are presented in form of modulus **(A)** and phase angle **(B)** in the frequency domain. The modulus of the input impedance of the peripheral VA-ECMO system is much higher than that of the central VA-ECMO system at frequency of 0 Hz (i.e., viscous resistance, which determines the relationship between mean blood pressure and flow rate), however, at higher frequencies, their relative magnitude changes alternatively with frequency. Note that the rotation speed of pump is set at 5,400 rpm for the two VA-ECMO systems.

The central and peripheral VA-ECMO modalities had similar effects on arterial blood pressure characterized by elevated pressure level and attenuated pulsation, but induced differential changes of blood flow waveforms in some arteries, especially those distal to the aortic arch (see Figure 4). The elevation of blood pressure and attenuation of pressure pulsation can be intuitively perceived since the ECMO pump provides additional power to drive the circulation of blood and the flow rate through the pump is nearly constant. The differences between the two ECMO modalities with respect to their effects on arterial blood flow waveforms are related to the site of outflow cannulation. In the case of peripheral VA-ECMO, the blood oxygenated in the ECMO oxygenator is injected back to the native circulatory system via the right external iliac artery, and the injected blood stream splits into two parts flowing in two directions, one is the retrograde flow toward the proximal aorta, which interacts with the forward blood flow sourced from the heart to reduce the total forward flow or even reverse the direction of blood flow in the aorta, whereas the other one flows toward the peripheral arteries in the leg. In the case of central VA-ECMO, the oxygenated blood is injected into the aortic arch located closely to the outlet of the left ventricle, which benefits the increase of blood flow in all arteries without altering the original flow direction. In addition, given the same operating parameters, the central VA-ECMO induced a slightly larger increase in arterial blood pressure than did the peripheral VA-ECMO. This is due to the lower afterload (can be evaluated by input impedance) distal to the outflow cannula of the central VA-ECMO than that of the peripheral VA-ECMO (see Figure 11 for the comparison of input impedances in the frequency domain), which allows a larger amount of blood to flow through the central VA-ECMO system (e.g., 72.7 mL/s vs. 66.3 mL/s when the rotation speed of ECMO pump was set at 5,400 rpm under the ‘Cardiac Failure I’ condition). As a consequence, given the same operating parameters of ECMO, the level of oxygen partial pressure in the arterial tree was overall higher with central VA-ECMO support than with peripheral VA-ECMO support. Interestingly, the differences of oxygen partial pressure between arteries did not exhibit strong dependence on ECMO modality (see Figure 4). In addition, the sensitivities of $S_{a,O_{2}}$ and $S_{v,O_{2}}$ to variations in *ω* were higher with peripheral VA ECMO support than with central VA-ECMO support, especially when *ω* was low and oxygen intake and supply by the native cardiopulmonary system were severely impaired by cardiopulmonary failure (see Figures 5, 6). These findings indicate that the central and peripheral VA-ECMO modalities differ evidently with respect to their influences on blood flow waveforms in the arterial tree, differ moderately in terms of the elevation of arterial pressure and the sensitivities of $S_{a,O_{2}}$ and $S_{v,O_{2}}$ to *ω*, and have a comparable capability to deliver oxygenated blood to major arteries. It should be noted however that the distribution of oxygenated blood sourced from the ECMO system in the arterial system may be considerably altered by some arterial diseases not considered in our study. For instance, a severe aortic stenosis may significantly impede the delivery of ECMO-oxygenated blood to vessels proximal or distal to the stenosis and thereby may augment the differences between the two ECMO modalities in supplying oxygenated blood to certain arteries.

Quantitative comparisons of model-simulated blood oxygen indices (i.e., saturation and partial pressure) in the right radial artery (where the status of blood oxygenation is usually monitored to assess the effect of ECMO support in clinical practice) with those in arteries supplying blood to important organs/tissues revealed that discrepancies were observable but small in magnitude (see Figures 7, 8). In particular, when the correctness of diagnosing hypoxemia in some important organs/tissues (e.g., brain, myocardium, and kidney) using radial arterial oxygen indices was evaluated, no obvious misdiagnosis or missed diagnosis was identified. Such phenomena held true for both central and peripheral VA-ECMO supports. These findings imply that blood oxygen indices measured at the right radial artery can serve as a reference for assessing the risk or severity of hypoxemia in major organs/tissues no matter what VA-ECMO modality is adopted, at least under the simulated pathological conditions in the present study.

Overloading of the left ventricle (LV) is another important issue of concern in clinical use of ECMO (Lorusso et al., 2021). The results of our numerical study for a selected pathological condition (i.e., Cardiac Failure I) showed that the central venous pressure (CVP) and LV end-diastolic volume (LVEDV), which are often measured to assess the severity of LV overloading in clinical settings, increased progressively with the increase in ECMO pump rotation speed (*ω*) (see Figure 9C). Such phenomena were accompanied by a progressive right upper shift of the LV P-V loop along with a rapid decrease in stroke volume (see Figure 9B), indicating that increasing *ω* significantly increases the diastolic and systolic myocardial stresses and inhibits the pumping function of the LV. In addition, aortic valve opening was intermittently interrupted when *ω* was high (>5,400 rpm) (see Figure 9A), under which condition CVP also increased over the threshold (>12 mmHg) for judging moderate LV overloading (Lorusso et al., 2021). Mechanisms underlying these observations are systemic hemodynamic changes caused by the competition between the ECMO system and the native cardiopulmonary system in delivering blood from the venous side to the arterial side. Increasing *ω* allows the ECMO system to intake a larger portion of venous blood, which, after being pressurized and injected back to the arterial system, raises LV afterload to inhibit cardiac emptying thereby aggravating cardiopulmonary congestion. It is interesting to remark that as the ECMO system operates under low *ω* conditions, the better preserved cardiac pumping function may partly retard the increase in venous pressure as indicated by the moderate increase in CVP with *ω* when *ω* < 4,800 rpm. Our additional numerical study for another pathological condition with more severe cardiac dysfunction (i.e., Cardiac Failure II) further showed that the threshold of *ω* corresponding to moderate LV overloading decreased to 4,100 rpm, indicating that the threshold rotation speed of ECMO pump depends strongly on the residual cardiac pumping function.

The theoretical findings may have some clinical implications. Under the simulated pathological conditions characterized by severe cardiac failure with or without concomitant pulmonary failure, the peripheral VA-ECMO modality is not significantly inferior to the central modality with respect to the delivery of oxygenated blood to major organs/tissues, and hence may be a reasonable choice given its advantage of relatively low invasiveness and complexity of artery cannulation. On the other hand, peripheral VA-ECMO support induces marked changes in blood flow patterns in the arterial system, such as reversed blood flow in the aorta, which might prevent its application to patients with aortic dissection because the retrograde flow will remarkably alter the flow patterns and biomechanical forces in the false lumen (Tse et al., 2011; Xu et al., 2018; Armour et al., 2020; Armour et al., 2022), which might increase the risk of further aggravation or even rupture of dissection (Schmidt et al., 2015). The comparable values of computed oxygen indices in the right radial artery and other arteries under various pathological conditions provide theoretical evidence supporting the clinical speculation that assessment of blood oxygenation via the right arm would reflect cerebral, and likely coronary, oxygenation (Krishnan and Schmidt, 2019). The numerical results regarding the severity and mechanisms of LV overloading associated with VA-ECMO support highlight the importance of taking into account patient-specific cardiac function in the assessment and management of LV overloading in clinical practice.

The study is subjected to certain limitations. One limitation is the theoretical nature of the study, which determines that the presented findings only provide insights for understanding the general characteristics of cardiovascular-ECMO coupling rather than precisely guiding the use of ECMO in specific patients. Theoretically, our model, if personalized to specific patients, could be applied to develop individualized ECMO treatment plans. Nevertheless, personalization of the model could be challenging since a large amount of clinical data required for parameter calibration should be measured prior to operation, which would significantly increase the complexity and cost of preoperative patient assessment. Another limitation is that our model did not incorporate the mechanisms of vascular responses to altered hemodynamic conditions and blood oxygenation associated with ECMO support, which will act to regulate arterial pressure and end-organ perfusion under *in vivo* conditions (Bělohlávek et al., 2010). Moreover, in the case of peripheral VA-ECMO, implanting the outflow cannula into the femoral and iliac arteries reduces the effective lumen areas of the native arteries, which will severely impede the blood flow directed from the outlet of the outflow cannula toward distal arteries in the leg (e.g., right anterior tibial artery) (Nezami et al., 2021). The effect was however ignored when we modeled peripheral VA-ECMO, which may explain why evident hypoxemia in the right anterior tibial artery was not predicted in our study, although ischemia and hypoxemia in the distal portion of the cannulated leg are common complications in patients supported by peripheral VA-ECMO (Patton-Rivera et al., 2018). To solve the problem, more sophisticated methods should be developed to incorporate the local hemodynamic effects of ECMO outflow cannulation into the modeling of systemic hemodynamics. Finally, it is worth noting that in the present study the severities of cardiac failure or cardiopulmonary failure were set to be extremely high in order to represent the critical clinical scenarios strongly indicating the initiation of VA-ECMO support (Lorusso et al., 2021), which implies that the applicability of the presented findings might be limited if pathological conditions deviate largely from the simulated ones. Some previous studies (Stevens et al., 2018; Feiger et al., 2020) have revealed that in patients with respiratory failure the supply of well-oxygenated blood sourced from the ECMO to the upper body (especially the brain) is affected considerably by the site of artery cannulation especially when the residual cardiac stroke volume is relatively large, which was however not evident from our numerical results. Although underlying reasons remain unclear, we speculate that the low residual cardiac contractility (≤20% of the normal value) assigned to our model, which remarkably reduces the relative contribution of cardiac pumping function to total blood circulation, may be a major contributing factor. In addition, unlike most open-loop models in the literature, the inlet and outlet boundary conditions of the arterial tree in our model are not fixed or artificially prescribed but spontaneously generated by numerical simulation of hemodynamic interaction and gas transport/exchange in the entire cardiopulmonary-ECMO system. Such differences in modeling method might partly account for the discrepancies between our results and previous findings, although how and to what extent modeling methods would affect the numerical results and associated findings would deserve further investigations.

## 5 Conclusion

A computational model has been built to quantitatively investigate VA-ECMO support-induced changes in hemodynamic variables and blood gas indices in cardiovascular systems suffering from severe cardiac or cardiopulmonary failure. It was found that under the model-simulated pathological conditions the central and peripheral VA-ECMO modalities had a similar capacity of oxygen supply while differential influences on arterial blood flow, and that blood oxygen indices in the right radial artery did not differ evidently from those in other arteries under both central and peripheral VA-ECMO supports. These findings imply that peripheral VA-ECMO, given the relatively low invasiveness and complexity of artery cannulation, may be a reasonable choice if improvement of blood oxygenation is the major demand, but its application to patients with hemodynamics-sensitive aortic diseases should be cautious in consideration of the marked alterations of blood flow patterns. In addition, improving blood oxygenation by increasing the rotation speed of ECMO pump was accompanied by a progressive increase in LV load and the critical rotation speed at which clinically significant LV overloading would occur was dependent on the residual cardiac contractility, which highlights the importance of taking into account patient-specific cardiovascular conditions in order to optimize the use of ECMO.

## Data Availability

The original contributions presented in the study are included in the article/Supplementary Material, further inquiries can be directed to the corresponding author.
